# Construction of a Novel Female Sterility System for Hybrid Rice

**DOI:** 10.3389/fpls.2021.815401

**Published:** 2022-02-03

**Authors:** Wei Li, Xiaoqiong Guo, Wenbin Wu, Weilin Yu, Shichuan Li, Di Luo, Tianjie Wang, Qian Zhu, Lijuan Chen, Dongsun Lee

**Affiliations:** ^1^Rice Research Institute, Yunnan Agriculture University, Kunming, China; ^2^State Key Laboratory for Conservation and Utilization of Bio-Resources in Yunnan, Yunnan Agricultural University, Kunming, China; ^3^The Key Laboratory for Crop Production and Smart Agriculture of Yunnan Province, Yunnan Agricultural University, Kunming, China

**Keywords:** female sterility, *fst* line, sexual propagation, transgene-free, FM-line system, hybrid rice

## Abstract

The main constraints of current hybrid rice technology using male sterility (MS) are the low yield and high labor costs of hybrid rice seed (HRS) production. Therefore, there is an urgent need for innovative new hybrid rice technology. Fortunately, we discovered a unique spontaneous sporophytic *female-sterile* rice mutant controlled by a single recessive locus in the nucleus. Because *female-sterile* mutant lines cannot produce any selfed-seeds but their pollen has totally normal functions, female sterility (FS) lines may be considered ideal pollen donors to replace the female-fertile pollen donor parent lines currently used in the HRS production. In this study, a genetically engineered FS-based system was constructed to propagate a pure transgene-free FS line using a bentazon herbicide screening. Additionally, the ability of the FS + MS (FM)-line system, with mixed plantings of FS and MS lines, to produce HRS was tested. The pilot field experiment results showed that HRS of the FM-line system was more efficient compared with the conventional FS to MS strip planting control mode. Thus, this study provides new insights into genetic engineering technology and a promising strategy for the utilization of FS in hybrid rice.

## Introduction

Rice (*Oryza sativa* L.) is an important staple food for more than one-half of the world’s population (Rao et al., 2018). However, rice cultivation still faces many challenges, such as a decrease in the farming labor force and shortages of land and water resources (Van Nguyen and Ferrero, 2006; Cheng et al., 2007). Generating hybrid rice lines, which have increased rice yields and improved tolerance levels to abiotic stresses compared with inbred rice lines, is one of the most successful applications of heterosis in the practice of agriculture and an efficient way to ensure food security (Kempe and Gils, 2011; Kim and Zhang, 2018; Wang and Deng, 2018).

To utilize hybrid vigor, two commercially viable hybrid systems utilizing cytoplasmic-genetic male sterility (CMS) and photoperiod- and thermo-sensitive genic MS genes, named as the 3-line and 2-line hybrid breeding systems, respectively, which produce first- and second generations of hybrid rice, respectively, have been developed and commercialized successfully in China (Yuan, 1966; Shi, 1985; Deng et al., 1999). In addition, a third-generation hybrid rice system has succeeded in propagating and utilizing recessive nuclear MS lines using a transgenic construct-driven non-genetically modified (GM) system called seed production technology (Chang et al., 2016; Wu et al., 2016; Basnet et al., 2019). Such a genetically engineered MS system has the ability to propagate non-transgenic MS seeds for hybrid rice seed (HRS) production and to overcome the intrinsic problems of the first two generations of hybrid rice systems (Wu et al., 2016; Fox et al., 2017; Zhang et al., 2018; An et al., 2019, 2020; Wan et al., 2019; Zhu et al., 2019; Song et al., 2020).

However, the benefits of the current first to third generations of hybrid rice systems are countered by increased seed production costs that result from complex HRS production procedures in the conventional strip planting mode (Zhang et al., 2017). For instance, the MS line, as the female parent, and the pollen donor line, as the male parent, should be transplanted and harvested separately. In addition to being a time-consuming, labor-intensive, and complicated process, it is also impossible to ensure that male plants have been completely removed and cannot contaminate the real hybrids (Kim et al., 2007). Therefore, using a female sterility (FS) line as the male parent has been proposed as the ideal seed production tool in combination with the current hybrid rice systems, we designed a novel HRS production system, with an FS + MS (FM)-line system, making it unnecessary to eliminate them after pollination during HRS production, in which the FS line acts as the pollen donor to replace the female-fertile pollen parent lines that cannot produce any self-pollinating or out-crossing seeds. This facilitates the high-quality and efficient mechanization of HRS production (Lee et al., 2002, 2013a; Chen et al., 2012).

Until now, only a few *FS* mutant genes, such as the *fst* (Lee et al., 2002, 2013a), *ptb1* (Li S.C. et al., 2013; Xia et al., 2019), and *fsv1* (Liu et al., 2019; Mao et al., 2021), have been identified in rice. However, for the commercial production of hybrids, FS acting as the pollen donor parent is only feasible if the FS line is completely sterile. Fortunately, we identified a B_*sister*_-group MADS-box gene, *FEMALE-STERILE* (*FST*) (GenBank: DQ004266.1), and the spontaneous *fst* mutant plants are completely sterile with normal anther development and viable pollen (Lee et al., 2002; Lee et al., 2013a).

How to multiply pure FS lines derived from the *fst* mutant efficiently and economically on a large scale needed to be determined. Several strategies for multiplying *fst* plants have been reported previously. One involved an asexual propagation technique, such as rice dormant bud regeneration (Lv et al., 2013) and rapid tiller propagation (Li S.C. et al., 2021). Another strategy is the development of a third-generation hybrid rice system (Xia et al., 2019). Furthermore, the most straightforward way to screen for sterile plants is to use a herbicide (Perez-Prat and Van Lookeren Campagne, 2002), as in the Bayer’s Seedlink (Kempe and Gils, 2011) and the new two-line (Kim et al., 2007) systems, but public concern regarding GM systems restricts the utilization of this approach. Consequently, there is a strong incentive to develop a practical and fully mechanized operation that generates non-GM HRS.

As previously reported, RNA interference (RNAi) generated knock-downs of *CYP81A6* gene may render rice plants susceptible to bentazon (BTZ*^S^*), and this BTZ*^S^* trait can be used as a marker trait to select lethal transgenic seedlings (Pan et al., 2006; Wang et al., 2012; Lu et al., 2017; He and Zhao, 2020). In this study, we chose to fundamentally redesign the target region in the conserved domain of *CYP81A6* to construct a new BTZ*^S^* lethality gene–RNAi expression cassette (BTZ-RNAi). A genetically engineered FS system for pure *fst* lines propagation was constructed by transforming an *fst* plant with the transgene *FST* (*FST^T^*) for the restoration of female fertility linked with the BTZ-RNAi cassette to create lethal transgenic female-fertile plants that could be killed by spraying the herbicide BTZ. We hypothesized that if the BTZ-RNAi cassette was closely linked to *FST^T^*, then the activity and presence of the T-DNA in the transgenic plants would confer the BTZ*^S^* phenotype and restore female fertility. Subsequently, we also tested a novel FM-line system for HRS production, with mixed plantings of FS and MS lines in different ratios and modes, which would facilitate the high-quality and efficient mechanization of HRS production.

## Materials and Methods

### Plant Materials and Growth Conditions

The *japonica* rice (*O. sativa* ssp. *japonica*) variety Ilmibyeo and the homozygous *fst* line Yunling319FS, containing the *Rf-1* gene, were used in this study. All the plants were planted in a greenhouse of the Rice Research Institute, Yunnan Agricultural University, Kunming (25.1°N, 102.7°N, 1,950 masl). In summer, the plants were grown in the greenhouse under natural conditions. In winter, the plants were grown in the greenhouse maintained at average day and night temperatures of over 30 and 20°C, respectively, with 12-h light/12-h dark cycles.

### Construction of Plant Expression Vectors

To construct the expression vector AT72 (*p35S:BTZ-RNAi*), a 304 bp fragment of the conserved domain of the BTZ*^S^* lethality gene *CYP81A6* (*Os.11193*) was amplified by polymerase chain reactions (PCR) from “Nipponbare” seedling stage leaf blade cDNA using the primer pair BTZRi-F/R (Supplementary Table 1). The fragment was then inserted into the dTA3 vector to form a hairpin structure, and an intron fragment containing 85 bp was used as the linker, which was released from the dTA3 vector by digestion with *Sal*I. The hairpin structure was introduced into a *Sal*I-predigested HPE203 binary vector between the 35S promoter and the nopaline synthase (NOS) terminator.

To produce the *FST^T^* gene expression cassette, we first amplified a 549 bp fragment from cDNA of “Nipponbare” taken at 5 days after pollination using the primer pair FT1-F/R (Supplementary Table 1) and a 647 bp fragment from “Nipponbare” genomic DNA using primer pair FT2-F/R (Supplementary Table 1). The upstream region of *FST* (2,289 bp) was amplified using the primer pair FTP1-F/R (Supplementary Table 1) from “Nipponbare” genomic DNA, and the NOS terminator fragment (314 bp) was amplified using the primer pair NOS1-F/R (Supplementary Table 1) from the HPE203 binary vector. These four fragments were fused together using overlapping extension PCR and inserted into the dTA3 vector. The obtained plasmid vector was digested with *Bsm*BI, and the *FST^T^* gene expression cassette (3,704 bp) was recovered. This was then cloned into the AT72 vector, which had been predigested with *Eco*RI and *Xba*I. This resulted in the expression vector AT73 (*pFST:FST//p35S:BTZ-RNAi*) (Supplementary Figure 1). AT72 and AT73 constructs were confirmed by sequencing before being transformed into *Agrobacterium tumefaciens* strain EHA105. Hygromycin resistance gene (*Hyg^R^*) is present on the backbone of the HPE203 binary vector, which allows for the subsequent selection of transgenic calli and plants using hygromycin B.

### *Agrobacterium*-Mediated Rice Transformation

The AT72 construct was transformed into rice calli, which were induced from mature embryos of the *japonica* rice line Ilmibyeo, and the construct AT73 was introduced into the young panicle- or anther-derived calli of the homozygous *fst* line Yunling319FS.

To obtain mature embryo-derived calli, mature seeds were dehusked and sterilized by incubation in 70% ethanol for 3 min and then in 1.5% (w/v) NaClO for 30 min with shaking. They were washed with sterile water five times, and the seeds were cultured on the N6D medium (N6 with 30 g/L sucrose, 2 mg/L 2,4-dichlorophenoxyacetic, and 2.4 g/L phytagel, pH 5.7) at 30°C in darkness. After 3 weeks, proliferated calli were subcultured on fresh N6D medium for 3 days and then used for transformation.

To obtain the anther-derived calli, rice panicles at the booting stage were collected and incubated at low temperature (10°C) for 10 days under dark conditions. After a cold pretreatment, the anthers were cultured on the CM6 medium (N6 with 70 g/L sucrose, 1 g/L yeast extract, 0.2 mg/L 2,4-dichlorophenoxyacetic, 1 mg/L naphthaleneacetic, and 10 g/L agar, pH 5.7) under aseptic conditions and then incubated in the dark at 30°C. At 3 weeks after inoculation, calli growth was observed from anthers, and the formed calli were sub-cultured on fresh N6D medium for another 3 days before being used for transformation.

To obtain the young panicle-derived calli, young panicles with spikelets were placed on the surface of N6D medium under aseptic conditions and incubated for 3 weeks in the dark at 30°C. Proliferating calli were subcultured on fresh N6D medium for another 3 days and were then used for transformation.

The constructs AT72 and AT73 were transformed independently into rice calli using *Agrobacterium tumefaciens*. Transgenic calli and plants with *Hyg^R^* were selected and subsequently transplanted.

### Identification of Transgenic Plants by Leaf PCR

Hygromycin resistance gene was employed as a selectable marker, and the transformants were screened by PCR amplification using standard amplification conditions (35 cycles of 20 s at 94°C, 20 s at 58°C, and 40 s at 72°C) with the following primer pair HpHmMAS2-F/R (Supplementary Table 1). The PCR products were examined by agarose gel electrophoresis.

### Phenotypic Identification and Microscopy

Plants and seeds were photographed using a Nikon D7500 digital camera. The seed setting rate (SSR) was estimated as the filled grain number per panicle divided by the total number of grains per panicle that includes the empty grains. The traits were measured as the mean ± SD (*n* = 10), and values of *p* were based on *t*-tests performed using R software (version 4.0.0^1^).

### Greenhouse Evaluation of Sensitivity to Bentazon

Tillers of T_0_ plants were separated and grown as two sub-plants. One normally growing sub-plant of each T_0_ plant was treated with a BTZ solution (1,200 mg/L) by spraying until droplets were visible on the leaves. At the three-leaf stage, the T_1_ seedlings were sprayed with BTZ solution (1,200 mg/L) until droplets were visible on the leaves. BTZ (48% solution) was obtained from Jiangsu BASF Limited (Jiangsu, China). Before the herbicides were sprayed on the rice plants, the field was drained to remove any field surface water. Three replicates of each rice line were evaluated for BTZ*^S^*.

### Isolation of the Flanking Sequence of T-DNA

The flanking sequence of T-DNA was isolated by inverse PCR to analyze the integration feature of the AT73 construct on the rice genome. One microgram of rice genomic DNA was digested with restriction endonuclease *Acc*65I, *Kpn*I, or *Cfr*42I and then self-ligated with T4 DNA ligase. Subsequently, relinearization of the DNA by restriction endonuclease *Hand*III and PCR was carried out, in which 0.5 μl of the digested product was amplified with primer pair Inver1-F/R (Supplementary Table 1). The PCR was performed following the amplification system for *TaKaRa LA Taq* DNA polymerase provided by the manufacturer. Then, products of PCR were separated by electrophoresis, recovered, and sequenced. The sequences were analyzed by performing a Basic Local Alignment Search Tool (BLAST) search in the NCBI database and Rice Genome Annotation Project database to investigate the integration feature of the AT73 construct on the rice genome.

### Hybrid Rice Seed Production Based on the FM-Line System

To test pollen viability, hybrid vigor potential, and the commercial viability of T_1_ transgene-free FS plants from AT73 transformants, they were taken as the male parent and were crossed to the elite Dian-type CMS line Hexi42A by hand emasculation and hand pollination. The resultant F_1_ hybrids were grown in a greenhouse.

A pilot experiment for HRS production was conducted to test the application potential of the FS+MS (FM)-line system. The transgene-free FS line 319FS was taken as the male parent and was mixed-planted with the elite Dian-type CMS line H479A, which has a similar growth duration, for HRS production at three different mixed-planting ratios and three different cultivation modes. The three cultivation modes used were as follows: the conventional strip planting mode as the control treatment, in which the seedlings were grown 30 days and then transplanted separately with one row of the transgene-free FS line and five rows of the MS line; the random mixed-planting mode, in which the seedlings of the FS and MS lines were grown 30 days and then mixed and transplanted randomly with one seedling per hill manually in 1:3, 1:4, and 1:5 ratios; the hill mixed-planting mode, in which the seedlings were grown 30 days and then transplanted manually at four seedlings per hill, with each hill containing one plant of the transgene-free FS line and three plants of the MS line. The pilot field experiments were carried out on the experimental fields of Yuanyang, Yunnan Province (23.1°N, 102.4°N, 260 masl). The experimental plot area was 25 m^2^ for each treatment and was conducted using a random block design with three replications. At the maturity stage, the panicle SSR of the maternal plants was recorded from more than 100 panicles randomly in each plot. The results were measured as the means ± SDs of three replicates, and values of *p* were based on *t*-tests performed using R software (version 4.0.0).

## Results

### Construction of a Transgene Seedling Lethality System With Bentazon

**FIGURE 1:**
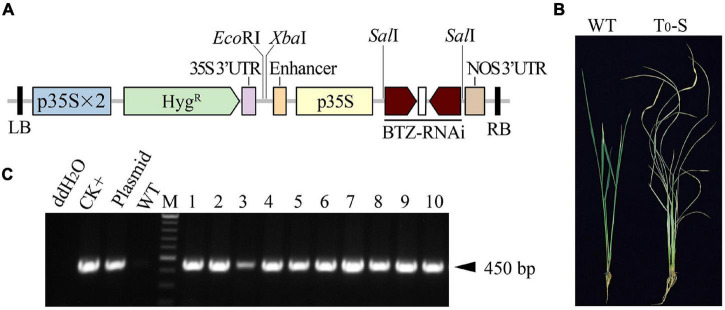
PCR screening and BTZ*^S^* testing of T_0_ transformants carrying the AT72 T-DNA fragment. **(A)** Schematic of the T-DNA region of the AT72 vector. p35S, cauliflower mosaic virus 35S promoter; p35S × 2, double 35S promoter; *Hyg^R^*, hygromycin resistance gene; 35S 3′-UTR, the 3′-UTR of the 35S promoter; BTZ-RNAi, the inverted repeat sequence of the 304 bp fragment of *CYP81A6*; LB and RB, left and right borders of the T-DNA, respectively; NOS 3′-UTR, the 3′-UTR of Nos; *Eco*RI, *Sal*I and *Xba*I are restriction enzyme sites. **(B)** Symptoms of a wild type (WT) Ilmibyeo sub-plant and a transgenic T_0_ BTZ*^S^* sub-plant (T_0_-S) 7 days after a BTZ foliar spray. **(C)** Agarose gel electrophoresis of PCR amplicons was created using *Hyg^R^*-specific primers. CK+: transgenic positive plant; Plasmid: HPE203 plasmid DNA with *Hyg^R^*; M: Thermo Scientific GeneRuler DNA Ladder Mix (SM0333); 1–10: T_0_ transgenic plants; UTR: untranslated region.

Because the BTZ*^S^* trait created by the RNAi knock-down of *CYP81A6* can be used to isolate transgene-free T_1_ rice plants, we chose to fundamentally redesign the target region of the conserved domain in *CYP81A6* (Supplementary Figures 2A,B) to construct a new BTZ-RNAi vector, AT72 (*p35S:BTZ-RNAi*) (Figure 1A). The calli derived from mature embryos of *japonica* rice c.v. Ilmibyeo were co-cultivated with *Agrobacterium tumefaciens* harboring the AT72 vector, and 10 BTZ*^S^* T_0_ plants (T_0_-S) were obtained from different calli (Figure 1B). Furthermore, these 10 plants were assessed by PCR using specific *Hyg^R^* primers, and a PCR band of the expected 450 bp was observed in all the T_0_ plant samples (Figure 1C).

**FIGURE 2:**
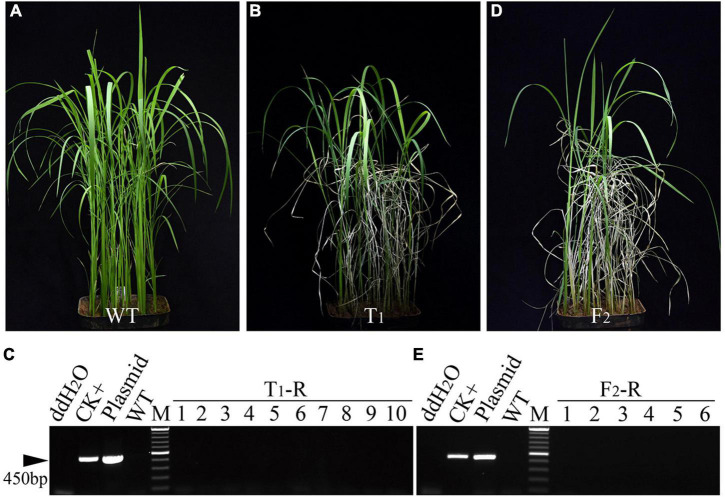
Screening of transgene-free plants from segregation progeny of independent transgenic T_0_ lines carrying the AT72 T-DNA fragment. **(A,B)** Symptoms of wild-type (WT) Ilmibyeo **(A)**, transgenic T_1_ progeny (#1) **(B)**. **(C)** PCR screening using *Hyg^R^*-specific primers for BTZ*^R^* plants (T_1_-R) from transgenic T_1_ progeny (#1). **(D)** Symptoms of F_2_ populations (#7) seedlings 7 days after a BTZ foliar spray. **(E)** PCR screening using *Hyg^R^*-specific primers for BTZ*^R^* plants (F_2_-R) from F_2_ populations (#7). CK+: transgenic positive plant; plasmid: HPE203 plasmid DNA with *Hyg^R^*; M: Thermo Scientific GeneRuler DNA Ladder Mix (SM0333). *Hyg^R^*, hygromycin resistance gene.

To evaluate the availability of the BTZ-RNAi expression cassette, a total of 500 plants of the T_1_ progeny derived from five T_0_-S lines were further tested (#1, #2, #3, #4, and #5). The four-leaf seedlings of the wild-type (WT) control (Figure 2A) and T_1_ progeny (Figure 2B and Supplementary Figure 3A) were tested by spraying them with BTZ. After approximately 7 days, some T_1_ seedlings showed the BTZ*^S^* phenotype and eventually died (T_1_-S), whereas other T_1_ seedlings remained resistant to BTZ (BTZ*^R^*) and were not visibly affected (T_1_-R). To evaluate the reliability of the transgene seedling lethality system, we used PCR with specific *Hyg^R^* primers to screen all the T_1_-R plants from the T_0_-S lines, and, as expected, no PCR bands were observed in any of the T_1_-R plants (Figure 2C and Supplementary Figure 3B). Thus, we found that the BTZ*^R^* plants were matched perfectly with the PCR results. To further investigate the heritability and stability of the BTZ-RNAi expression cassette in rice, T_0_-S line #3 was selected as the male parent and cross-pollinated with “Hexi 42A,” an elite CMS line. The two F_1_ *Hyg^R^* positive plants as assessed by PCR also exhibited the BTZ*^S^* phenotype, whereas the two F_2_ populations (#6 and #7)performed PCR using *Hyg^R^*-specific primers, but no fragments were amplified in the total 188 F_2_-R plants (Figures 2D,E and Supplementary Figure 3). Thus, the expression vector AT72 appears to enable the establishment of an effective and accurate transgene seedling lethality system for screening transgene-free plants by spraying them with BTZ.

### Development of a Genetically Engineered Female Sterility System Based on the Transgenic Seedling Lethal Cassette

**FIGURE 3:**
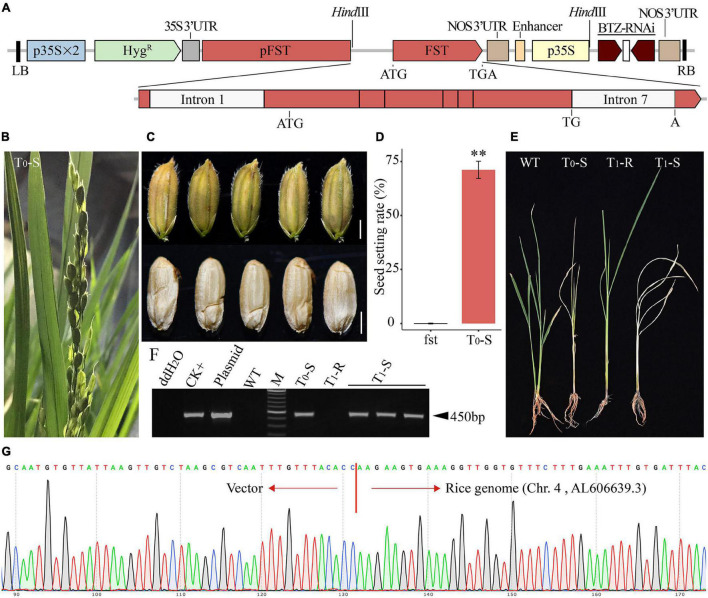
The phenotype of the genetically engineered FS line. **(A)** Diagram of the expression vector AT73. p35S, cauliflower mosaic virus 35S promoter; p35S × 2, double 35S promoter; *Hyg^R^*, hygromycin resistance gene; 35S 3′-UTR, the 3′-UTR of the 35S promoter; BTZ-RNAi, the inverted repeat sequence of the 304 bp fragment of *CYP81A6*; LB and RB, left and right borders of the T-DNA, respectively; NOS 3′-UTR, the 3′-UTR of Nos; pFST, the promoter of the *FST* gene. **(B)** A mature panicle of a transgenic complemented T_0_ BTZ*^S^* plant. **(C)** The mature seeds of a transgenic complemented T_0_ BTZ*^S^* plant (scale bar, 2 mm). **(D)** Characterization of SSR in an FS plant and BTZ*^S^* T_0_ line (T_0_-S). The data are presented as means ± SDs (*n* = 10), and a statistical analysis using *t*-tests indicated the significant differences (^**^p < 0.01). **(E)** Symptoms of a wild-type (WT) Ilmibyeo sub-plant and transgenic T_0_ and T_1_ sub-plants 7 days after a BTZ foliar spray. T_0_-S: T_0_ BTZ*^S^* plant; T_1_-R: T_1_ BTZ*^R^* plant; T_1_-S: T_1_ BTZ*^S^* plant. **(F)** Agarose gel electrophoresis results of PCR screening using *Hyg^R^*-specific primers. CK + : transgenic positive plant; Plasmid: HPE203 plasmid DNA with *Hyg^R^*; M: Thermo Scientific GeneRuler DNA Ladder Mix (SM0333). **(G)** Representative chromatograms of the transgene integration locus in T_0_ transgenic event AT73-28. SSR, seed setting rate.

Because *FST* is a sporophytic female fertility gene in rice, a hemizygous *FST^T^* in the *fst* mutant plant that can fully restore female fertility. The T_0_ transgenic plants (*FST^T^*/*fst*) were allowed to self-pollinate, and the resulting T_1_ progeny should show the segregation of transgenic female-fertile and transgene-free FS plants. To obtain all transgene-free FS seedlings from T_1_ progeny efficiently and economically, the linkage expression vector AT73 (*pFST:FST//p35S:BTZ-RNAi*) was constructed, containing the *FST^T^* expression cassette, which consisted of the 1.8-kb *FST* promoter, the *FST* coding sequence with the first and last introns and the BTZ-RNAi expression cassette (Figure 3A). The AT73 construct was transformed into the young panicle-originated or anther-derived calli of the homozygous *fst* line Yunling319FS using the *Agrobacterium tumefaciens*-mediated method to obtain hemizygous *FST^T^* T_0_ plants.

Based on the expected 3:1 ratio of BTZ*^S^* and BTZ*^R^* seedlings, one T_0_ transgenic event, AT73-28, was chosen as a representative for further study. The T_0_ plants (by tiller propagation) were allowed to self-pollinate and displayed dominant female fertility restoration, with a greater than 70% SSR under greenhouse conditions (Figures 3B–D), and the self-pollinated mature seeds presented almost the same phenotype as normal seeds (Figure 3C). Furthermore, BTZ*^S^* test results showed that the T_0_ plants (T_0_-S) died about 1 week later by spraying BTZ (Figure 3E), and a PCR analysis using *Hyg^R^*-specific primers confirmed that the T_0_ plant was transgenic (Figure 3F). The integration site of T-DNA borders in the T_0_ plant was analyzed by flanking sequence isolation. T_0_ plant AT73-28, which was analyzed three times by inverse PCR, gave the same location of integration, and T-DNA integration at 105,018 bp (GenBank Accession No. AL606639.3) at chromosome 4 in the rice genome (Figure 3G and Supplementary Figure 4).

In addition, all of the individual T_1_ plants of AT73-28 were analyzed by PCR using *Hyg^R^*-specific primers to determine whether they were transgenic plants or segregates without the transgene. The *Hyg^R^*-specific primers did not amplify any bands in some of the individual T_1_ plants (Figure 3F). Additionally, BTZ*^S^* test results showed that all of the positive transgenic T_1_ plants (T_1_-S: *FST^T^*/*fst* and/or *FST^T^*/*FST^T^*) died within approximately 7 days of being sprayed with BTZ, whereas the negative T_1_ segregates (T_1_-R: *fst/fst*) without the transgene survived as expected (Figure 3E).

Thus, the transgenic event AT73-28 appeared to produce heterozygotes (*FST^T^*/*fst*) of the *FST^T^* loci, and the presence of the T-DNA cassettes, such as the BTZ*-*RNAi, *FST^T^*, and *Hyg^R^* expression cassette (*pFST:FST//p35S:BTZ-RNAi*), was tightly linked in T_0_ plant. Therefore, this system may efficiently propagate the transgene-free FS line (*fst*/*fst*) by self-pollination of the genetically engineered FS line and transgene seedling lethality screening at the seedling stage in a greenhouse.

### Application of the FM-Line System to Hybrid Rice Seed Production

**FIGURE 4:**
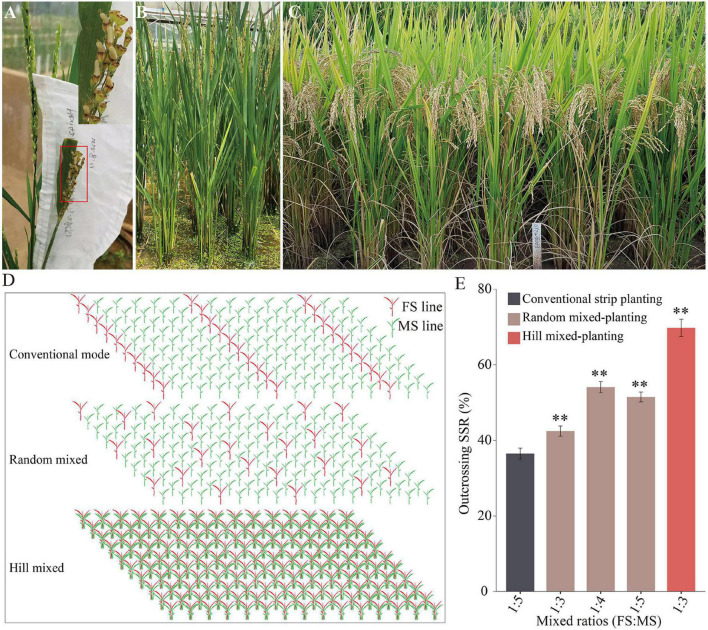
HRS production is based on the FM-line system. **(A)** The seed setting of the maternal ‘Hexi42A’ plant is pollinated by pollen from the transgene-free FS line 319FS. **(B)** Morphology of transgene-free FS line 319FS. **(C)** Plant-type and heterosis observations of the hybrid F_1_ generation of Hexi42A/319FS. **(D)** Diagram of the FM-line system using three different mixed-planting methods for HRS production. **(E)** Statistical data of the outcrossing SSR of the maternal plants in HRS production using the FM-line system. The data are presented as means ± SDs (*n* = 100), and a statistical analysis using *t*-tests indicated the significant differences (^**^p < 0.01). HRS, hybrid rice seed; SSR, seed setting rate.

Because the transgene-free T_1_ plants were completely FS, they could be cross-pollinated with MS plants for the mechanized production of HRS. The transgene-free FS line 319FS of the T_1_ plants (T_1_-R) from AT73-28 was used as the pollen donor to cross with a Dian-type CMS line “Hexi42A,” and their hybridization produced a normal SSR as expected (Figures 4A,B). The F_1_ hybrids were planted with proper management, and they exhibited the expected heterosis and ideal architecture with good performance (Figure 4C). Thus, the transgene-free FS line 319FS may be used as the pollen donor parent and applied to improve HRS production.

**FIGURE 5:**
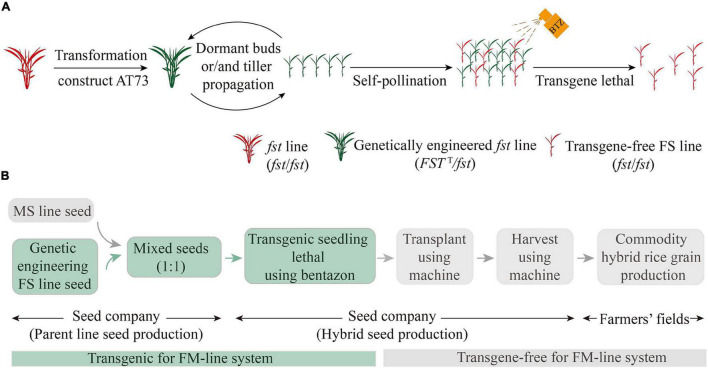
Schematic representation of the FM-line system process for the mechanization of HRS production. **(A)** The genetically engineered FS system to propagate transgene-free FS lines. The red-colored plants are non-transgenic or transgene-free; the green-colored plants are transgenic. **(B)** The FM-line system for mechanized HRS production. HRS, hybrid rice seed.

In a pilot experiment, we tested the mean outcrossing SSR of the MS plants by establishing three different FS line 319FS and MS line H479A mixed-planting modes (Figure 4D). There was a significant difference in panicle SSR of the MS plants compared with the conventional strip planting mode (Figure 4E). The mean outcrossing SSRs of MS plants from the random 1:3, 1:4, and 1:5 FS and MS lines’ mixed-planting modes were 42.45, 54.07, and 51.47%, respectively. In the hill 1:3 of FS and MS lines’ mixed-planting modes, the mean outcrossing SSR of MS plants was 69.77%, whereas that of the MS line of the conventional 1:5 FS and MS lines’ strip planting control mode was 36.49%. Thus, there is great potential for considering the FS lines as the ideal pollen donors to replace the female-fertile pollen parent lines (i.e., restorer lines) currently used in 2-, and 3-line systems of HRS production. Taken together, it was further illustrated that the genetically engineered FS system (Figure 5A) and FM-line system (Figure 5B), and mixed-plantings of FS and MS lines, greatly reduced labor for transplanting and harvesting separately male parents.

## Discussion

In the present study, the *fst* mutant did not produce any self-pollinating or outcrossing seeds, whereas it showed normal vegetative growth and pollen fertility. During the implementation of the hybrid rice breeding projects at Yunnan Agricultural University, over a dozen perennial FS lines containing the CMS restorer gene (*Rf-1*) (Komori et al., 2004) in different genetic backgrounds were bred via complex hybridization and self-breeding based on the *fst* mutant (Li S.C. et al., 2021; Yu et al., 2021; Zhu et al., 2021). Currently, the *de novo* development of FS lines in different restorer backgrounds using the CRISPR-Cas9 genome editing technology is underway (our unpublished data) which could y assist in developing more efficient FS lines to be broadly utilized for hybrid rice breeding. At present, to propagate a large number of plants and/or seeds of pure transgene-free FS line for facilitating the mechanization of HRS production, we designed a novel biotechnology-based genetically engineered FS system (Figure 5A) and FM-line system (Figure 5B).

The FM-line system is superior to the traditional HRS production method and other biotechnology-based systems in several aspects. First, relative to the other FS mutants, such as *ptb1* (Li S.C. et al., 2013) and *fsv1* (Liu et al., 2019; Mao et al., 2021), here, the FS lines were created by hybridization breeding and using a natural mutant *fst* (Lee et al., 2013a), and the plants propagated were completely sterile in different genetic backgrounds and under different environmental conditions. Second, compared with the current 2- and 3-line systems of HRS technology, the FM-line system facilitated the mechanization of high-quality and efficient HRS production using a mixed-planting and mixed-harvesting approach for FS and MS lines. Third, BTZ*^S^* test results confirm that FM-line system works as efficiently as other transgene sorting systems, such as fluorescence-assisted sorting method (Aliaga-Franco et al., 2019; Xia et al., 2019), weight-based seed sorting system (Wu et al., 2021), and anthocyanin-marker-assisted sorting system (He et al., 2020), but with the advantage of using the BTZ*^S^* trait to select lethal transgenic seedlings due to its simplicity, accuracy, and sensitivity. Finally, neither the commercial HRS produced using the transgene-free FS line resulting from the genetically engineered FS system nor the resulting commodity rice grain harvested from these hybrid plants contained transgene DNA and were, therefore, transgene-free in the FM-line system (Figure 5B). Furthermore, the genetically engineered *fst* lines (*FST^T^*/*fst*) propagation by dormant buds or tillers requires limited acreage in greenhouse or fields together with strict spatial or temporal isolation measures would minimize the chances of out-pollination (Figure 5A). The future perspect of commercial production of HRS using FS lines focuses on the state-of-the-art developments in the seed company under the government’s supervision.

In the FM-line system, other particular concerns are how to maintain and propagate a large quantity of the genetically engineered FS lines, such as the T_0_ line AT73-28. Prior studies have noted the importance of producing FS plants by asexual propagation based on rice dormant bud regeneration (Lee et al., 2013b) or iterative rapid tiller propagation (Li W. et al., 2021). During the T_0_ line propagation phase, rice dormant bud regeneration or rapid tiller propagation can multiply a large number of plants, and the T_1_ seeds were produced by T_0_ plant self-pollination. During the FS-line propagation phase, the transgenic seedlings can be selectively killed by BTZ spraying and those that remained alive represent transgene-free FS plants. Prerequisites for the FM-line system are the reliability and accuracy of transgene seeding lethality screening with BTZ. Our studies showed that all the transgenic rice plants in the T_0_, T_1_, and F_2_ populations could be selectively killed by BTZ spraying, suggesting that the BTZ-RNAi expression cassette had a high screening efficiency. Additionally, previous studies have established that transgenic rice plants can be selectively killed at 100% by BTZ spraying (Lin et al., 2008; Liu et al., 2012; Lu et al., 2017). Thus, transgenic oversight is applicable only to the genetically engineered FS lines’ cultivation and transgene-free FS line propagation, which requires limited acreage. Concerns regarding the evolution of herbicide resistance in natural variations can be buffered by using a rigorous screening by bentazon spraying during the different phases of HRS production. Careful analysis of the mixed FS and MS seedlings and F_1_ seeds for the transgene before their commercial production would minimize the potential risk of environmental contamination. In this context, we showed that the *fst* mutant and its derived lines can be used as transgene-free FS lines in the FM-line system. The *fst* mutation confers stable genic-controlled FS in both *indica* and *japonica* rice. Thus, the *fst* mutation has great potential in commercial HRS production. Our studies indicated that the *FST^T^* expression cassette in the AT73 construct was able to restore spikelet fertility (Figure 3D). Furthermore, the rice *FST* is a key regulator and plays multiple functions during ovule and early seed development (Yang et al., 2012; Yin and Xue, 2012; Lee et al., 2013a). While this study clearly demonstrated that the FM-line system is feasible, more detailed studies are required to optimize the *FST* gene expression cassette needed to generate the genetically engineered FS lines with desirable seed development and SSR.

Our pilot experiment’s results showed that different mixed-planting modes and ratios affect the yield of HRS production in the FM-line system, especially in the hill 1:3 mixed-planting mode, in which the mean outcrossing SSR of the MS line was almost double that of the conventional strip planting control mode (Figure 4E). For practical HRS production, the outcrossing SSR of MS plants should increase as a result of additional artificial supplementary pollination processes. Thus, further field experiments are required to establish the optimal proportion of FS and MS lines’ mixed-planting modes in combination with additional artificial supplementary pollination processes, which would facilitate the high quality and efficient mechanization of HRS production.

## Data Availability Statement

The original contributions presented in the study are included in the article/Supplementary Material, further inquiries can be directed to the corresponding authors.

## Author Contributions

DSL and LJC designed the experiments. WL and XQG performed the experiments, analyzed the data, and drafted the manuscript. QZ, WBW, WLY, SCL, DL, and TJW participated in performing the experiments. All authors reviewed and approved the manuscript for publication.

## Conflict of Interest

The authors declare that the research was conducted in the absence of any commercial or financial relationships that could be construed as a potential conflict of interest.

## Publisher’s Note

All claims expressed in this article are solely those of the authors and do not necessarily represent those of their affiliated organizations, or those of the publisher, the editors and the reviewers. Any product that may be evaluated in this article, or claim that may be made by its manufacturer, is not guaranteed or endorsed by the publisher.

## Footnotes

1
https://www.R-project.org/
