# Supplementary material for: Construction of a Novel Female Sterility System for Hybrid Rice
Source: Front Plant Sci. 2022 Feb 3;12:815401. doi: 10.3389/fpls.2021.815401 (PMC8850283; doi:10.3389/fpls.2021.815401)
Supplement: Supplementary file 1 [file Data_Sheet_1.docx]

**Supplementary Material**

**Supplementary Figure 1**


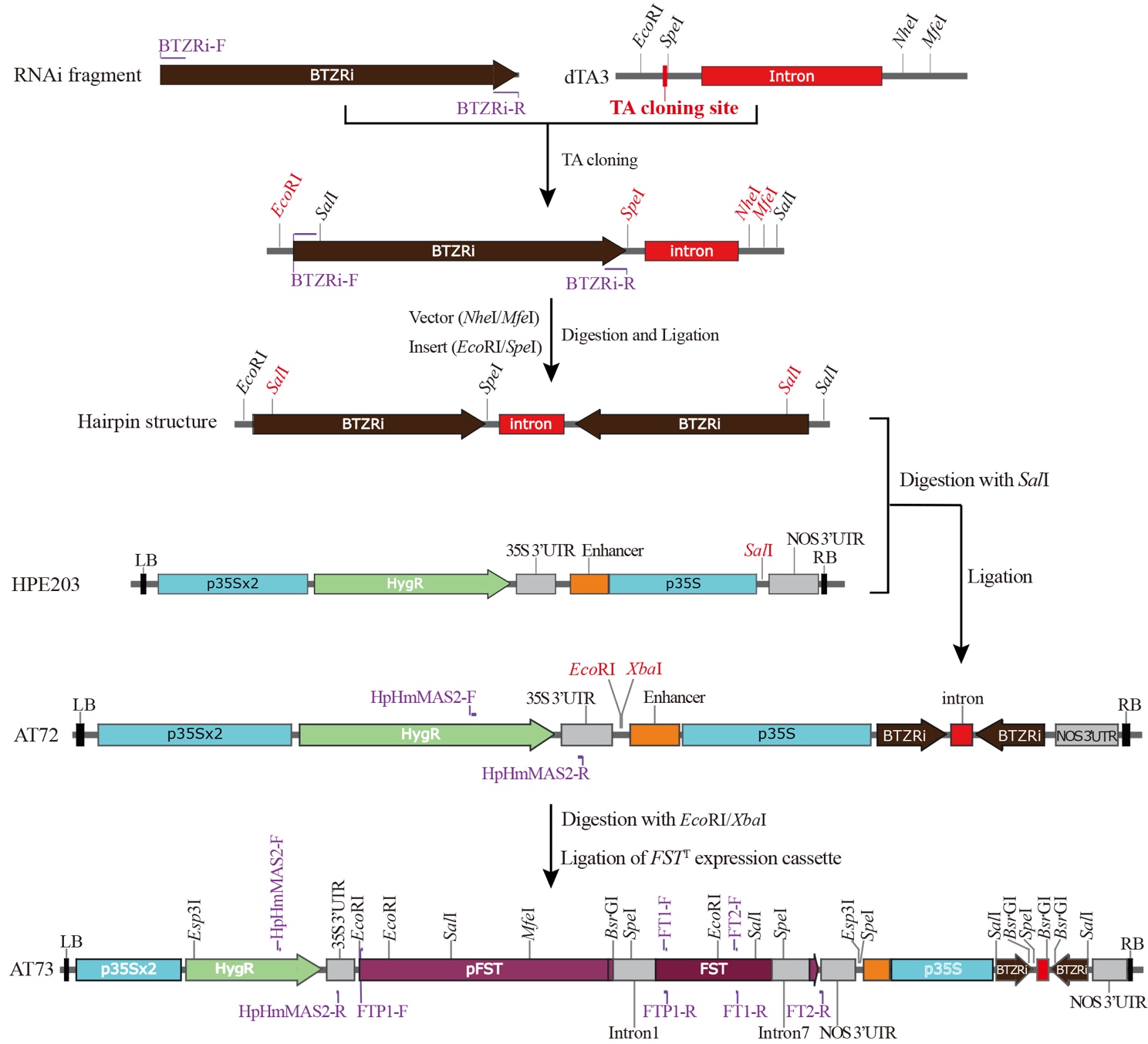


**Supplementary Figure 1.** Diagrammatic presentation of vector construction and primer positions.

**Supplementary Figure 2**

**
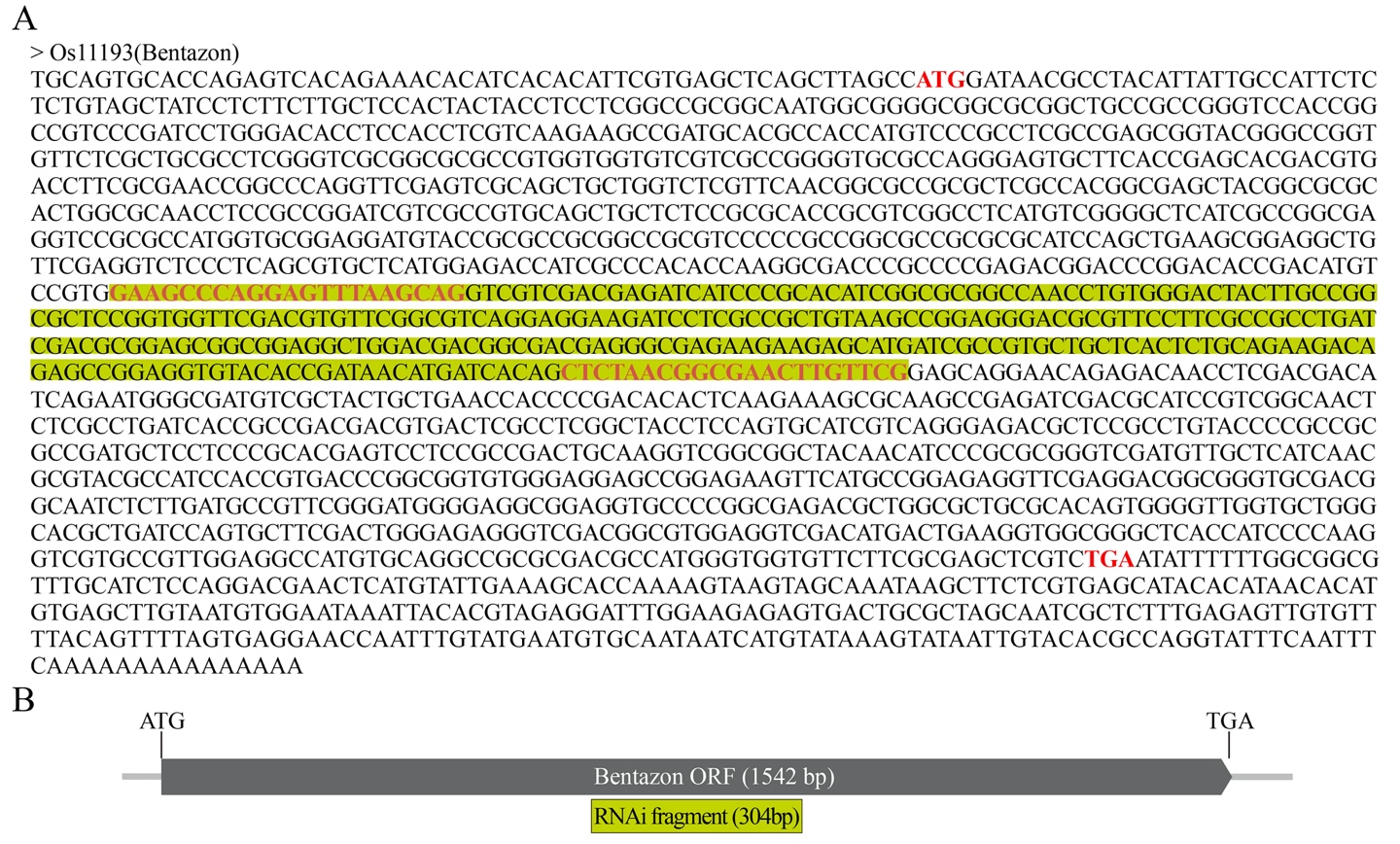
**

**Supplementary Figure 2.** Molecular cloning of the BTZ-RNAi expression cassette. **(A)** The CDS of the BTZ^S^ lethality gene *CYP81A6* (*Os.11193*) in this study. **(B)** Diagram depicting the BTZ^S^ lethality gene *CYP81A6* (*Os.11193*) open-reading frame (1,542 bp), the RNAi fragment (304 bp) and the cloning position for the BTZ-RNAi expression cassette.

**Supplementary Figure 3**


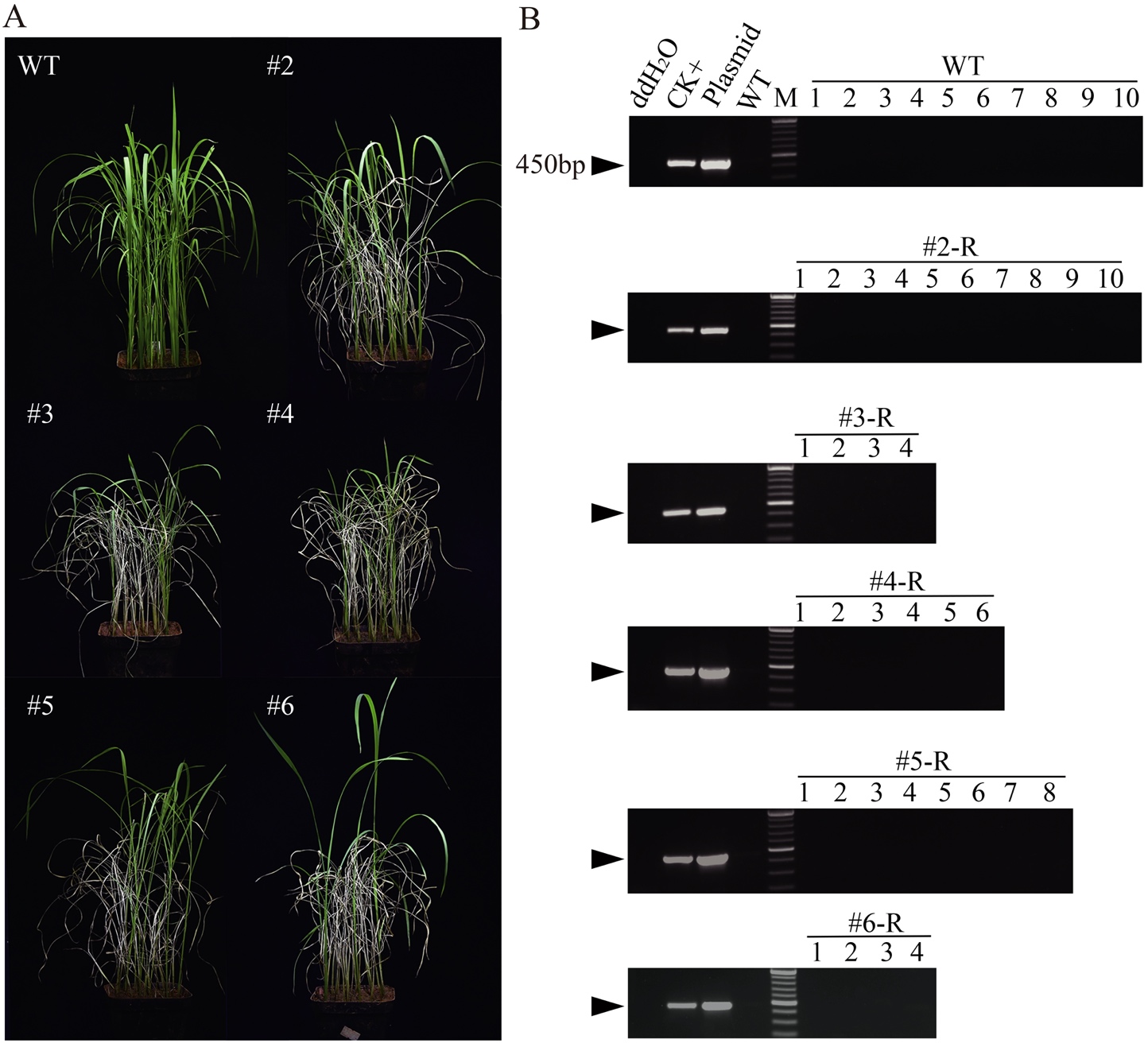


**Supplementary Figure 3.** Screening of transgene-free plants from segregation progeny of independent transgenic T_0_ lines carrying the AT72 T-DNA fragment. **(A)** Symptoms of wild type (WT) ‘Ilmibyeo’, transgenic T_1_ progeny (#2, #3, #4 and #5) and F_2_ populations (#6) seedlings at 7 days after a BTZ foliar spray. **(B)** PCR screening using *Hyg^R^*-specific primers for T_1_ BTZ^R^ plants (T_1_-R) and F_2_ BTZ^R^ plants (F_2_-R). CK+: transgenic positive plant; Plasmid: HPE203 plasmid DNA with *Hyg^R^*; M: Thermo Scientific GeneRuler DNA Ladder Mix (SM0333).

**Supplementary Figure 4**


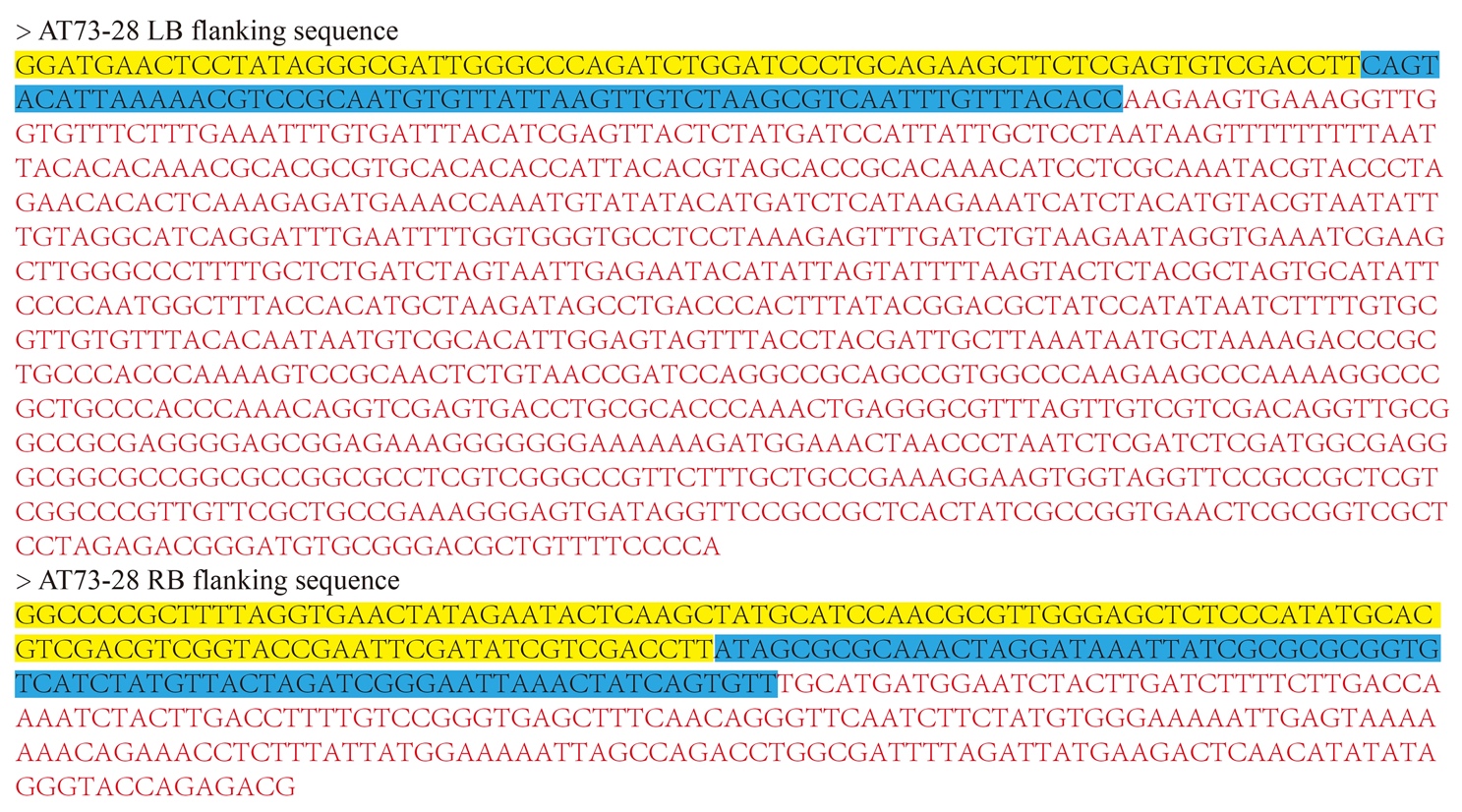


**Supplementary Figure 4.** ﻿The flanking sequence of T-DNA in T_0_ transgenic event AT73-28. Sequences with yellow background represent binary vecter sequences; sequences with blue background represent the LB (left border) sequences; sequences with green background represent the RB (right border) sequences; red font sequences represent rice genomic sequences.

**Supplementary Table 1**

**Supplementary Table 1. Primers used in this study.**

| Primer name | Primer sequence (5'-3') |
| --- | --- |
| FT1-F | GATCAGGAAGGTATAGCAGGCAT |
| FT1-R | CGCCTGATGATGGTTCTCGT |
| FT2-F | ACGAGAACCATCATCAGGCGGCG |
| FT2-R | GAACGATCGGGGAAATTTCCAAGTTACTCCTAG |
| NOS1-F | CTAGGAGTAACTTGGAAATTTCCCCGATCGTTC |
| NOS1-R | CTTCACTAGTGAGACGACACTGATAGTTTAATTCCC |
| FTP1-F | AACGTCTCGAATTCTAACAGCCAATGACCC |
| FTP1-R | CATGCCTGCTATACCTTCCTGATCGAG |
| BTZRi-F | GAAGCCCAGGAGTTTAAGCAG |
| BTZRi-R | CGAACAAGTTCGCCGTTAGAG |
| HpHmMAS2-F | GAGGTCGCCAACATCTTCTTCTG |
| HpHmMAS2-R | AGCGAAACCCTATAGGAACCCTAA |
| Inver1-F | CAGTACATTAAAAACGTCCGCA |
| Inver1-R | ATAGCGCGCAAACTAGGATAAA |
